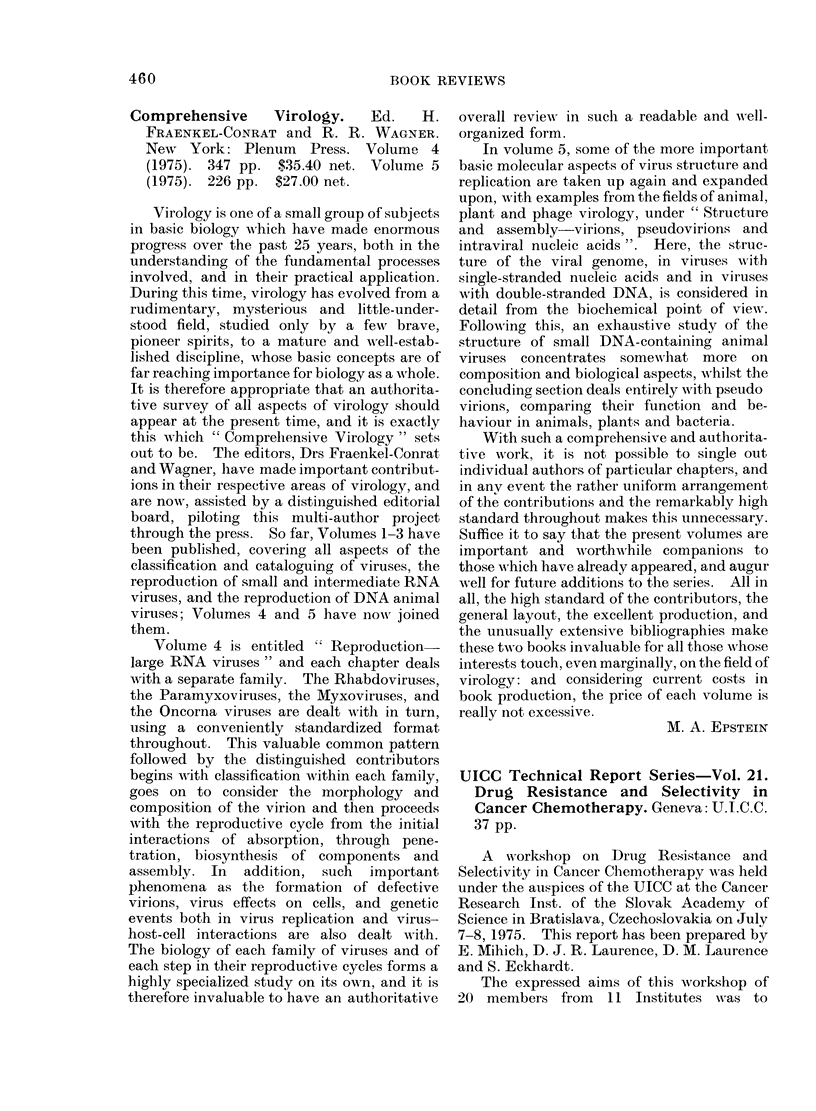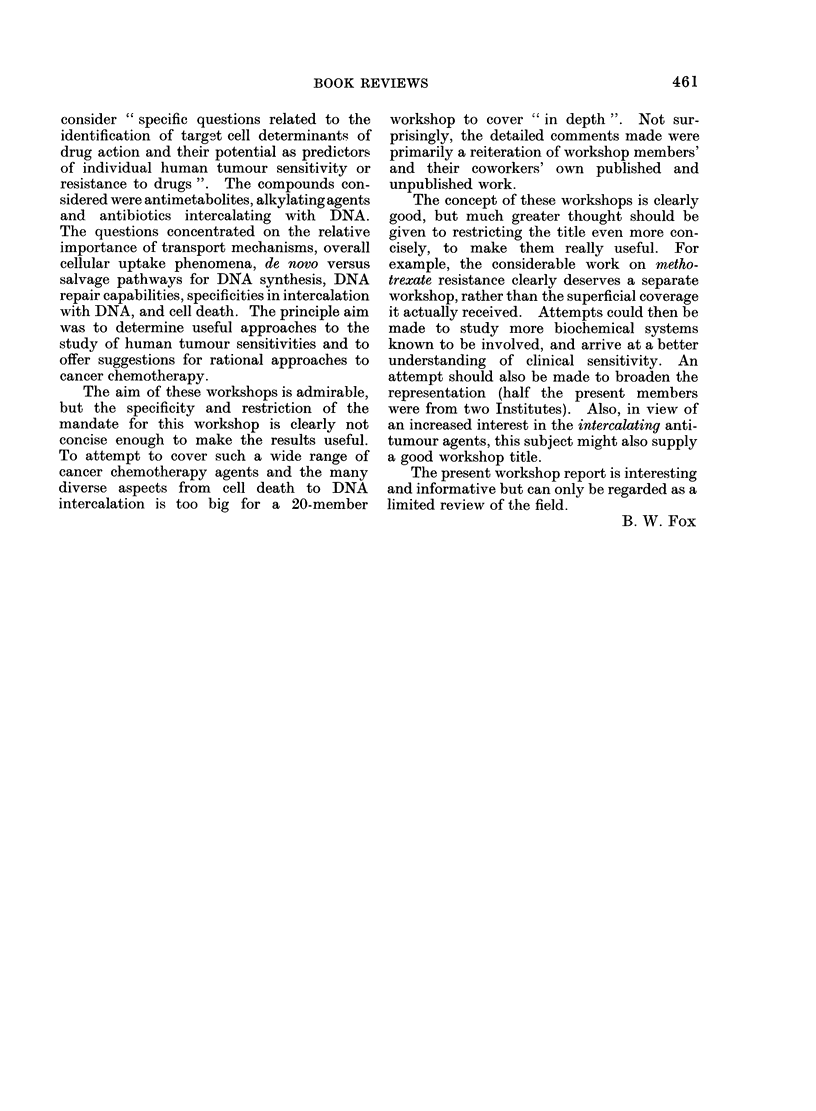# UICC Technical Report Series—Vol. 21. Drug Resistance and Selectivity in Cancer Chemotherapy

**Published:** 1976-10

**Authors:** B. W. Fox


**UICC Technical Report Series—Vol. 21. Drug Resistance and Selectivity in Cancer Chemotherapy.** Geneva: U.I.C.C. 37 pp.

A workshop on Drug Resistance and Selectivity in Cancer Chemotherapy was held under the auspices of the UICC at the Cancer Research Inst. of the Slovak Academy of Science in Bratislava, Czechoslovakia on July 7–8, 1975. This report has been prepared by E. Mihich, D. J. R. Laurence, D. M. Laurence and S. Eckhardt.

The expressed aims of this workshop of 20 members from 11 Institutes was to consider " specific questions related to the identification of target cell determinants of drug action and their potential as predictors of individual human tumour sensitivity or resistance to drugs ". The compounds considered were antimetabolites, alkylating agents and antibiotics intercalating with DNA. The questions concentrated on the relative importance of transport mechanisms, overall cellular uptake phenomena, *de novo* versus salvage pathways for DNA synthesis, DNA repair capabilities, specificities in intercalation with DNA, and cell death. The principle aim was to determine useful approaches to the study of human tumour sensitivities and to offer suggestions for rational approaches to cancer chemotherapy.

The aim of these workshops is admirable, but the specificity and restriction of the mandate for this workshop is clearly not concise enough to make the results useful. To attempt to cover such a wide range of cancer chemotherapy agents and the many diverse aspects from cell death to DNA intercalation is too big for a 20-member workshop to cover " in depth ". Not surprisingly, the detailed comments made were primarily a reiteration of workshop members' and their coworkers' own published and unpublished work.

The concept of these workshops is clearly good, but much greater thought should be given to restricting the title even more concisely, to make them really useful. For example, the considerable work on *methotrexate* resistance clearly deserves a separate workshop, rather than the superficial coverage it actually received. Attempts could then be made to study more biochemical systems known to be involved, and arrive at a better understanding of clinical sensitivity. An attempt should also be made to broaden the representation (half the present members were from two Institutes). Also, in view of an increased interest in the *intercalating* antitumour agents, this subject might also supply a good workshop title.

The present workshop report is interesting and informative but can only be regarded as a limited review of the field.

B. W. Fox